# Stakeholder views about the responsibilities of principal investigators in multicenter randomized controlled trials

**DOI:** 10.1177/17407745261417337

**Published:** 2026-02-23

**Authors:** Steven Joffe, Elizabeth F Bair, Katharine A Gleason, Deborah E Sellers, Sarah A McGraw, Cary P Gross, Donna T Chen, Eric G Campbell, Michelle M Mello

**Affiliations:** 1 Department of Medical Ethics & Health Policy, Perelman School of Medicine at the University of Pennsylvania, Philadelphia, PA, USA; 2 Bronfenbrenner Center for Translational Research, Cornell University, Ithaca, NY, USA; 3 The Hastings Center for Bioethics, Garrison, NY, USA; 4 MNW Consulting Group, LLC. Portland, OR, USA; 5 Department of Medicine and Cancer Outcomes, Yale School of Medicine, New Haven, CT, USA; 6 Center for Health Humanities & Ethics, University of Virginia School of Medicine, Charlottesville, VA, USA; 7 Center for Bioethics and Humanities, University of Colorado Anschutz Medical Campus, Aurora, CO, USA; 8 Department of Health Policy, Stanford University School of Medicine, Stanford, CA, USA

**Keywords:** Randomized clinical trial, principal investigator, research integrity

## Abstract

**Background/Aims:**

Clinical trials are commonly believed to benefit from the involvement of an academic principal investigator who accepts responsibility for design, conduct, and reporting. Little evidence exists, however, about the importance that diverse stakeholders assign to the principal investigator’s role in leadership of trials. Furthermore, few studies have examined whether and how beliefs about the role of the principal investigator might vary by funding source.

**Methods:**

We conducted parallel Delphi panel surveys with seven stakeholder groups (principal investigators, patient advocates, journal editors, public funders, industry representatives, United States Food and Drug Administration officials, and clinical trial cooperative-group chairs) to assess the extent to which respondents believed leadership of a multicenter randomized controlled trial by an academic principal investigator to be important, considering publicly and industry-funded trials separately. We then surveyed an international sample of principal investigators (N = 92) who had recently published a multicenter randomized controlled trial in a high-impact general medical, oncology, cardiovascular, or psychiatry journal to assess their normative views on the importance of the academic principal investigator in leading both publicly and industry-funded trials.

**Results:**

Several patterns emerged from the Delphi panel surveys. First, panelists viewed involvement of an identified academic principal investigator as most important at the design and planning and the interpretation and dissemination phases of a trial, as compared with the implementation and data collection phase. Second, panelists generally viewed involvement of an identified academic principal investigator as more important in publicly funded than in industry-funded trials. Finally, panelists representing industry stakeholders and United States Food and Drug Administration officials viewed involvement of an identified academic principal investigator as less important, especially for industry-funded trials, than did other groups. Respondents to the normative principal investigator survey generally endorsed the importance of academic principal investigators in leading multicenter randomized controlled trials, both overall (median rating 6 on the 0–6 point scale) and for trial-specific tasks. Both overall and with respect to specific tasks, however, respondents viewed an academic principal investigator’s leadership as more important when considering publicly funded as compared with industry-funded trials.

**Conclusion:**

Although members of most stakeholder groups participating in Delphi surveys view involvement of an academic principal investigator with overall responsibility for a multicenter randomized controlled trial as very important, there are notable differences depending on the respondent’s perspective, the specific trial-related task, and the source of funding for the trial. In addition, principal investigators generally view an academic principal investigator as more important to the validity of a publicly funded multicenter randomized controlled trial than to the that of an industry-funded trial. These findings highlight the need to clarify the real-world practices of clinical trial leadership across diverse settings and to assess how those practices align with widely shared norms.

## Introduction

Responding to concerns about the integrity and ethics of clinical research, stakeholders in the research enterprise, including journals, academic medical centers, and public and for-profit sponsors, have devised mechanisms to promote accountability for the conduct of clinical trials. Concerns are heightened in the setting of industry-sponsored trials, given evidence of problematic practices and potential bias in many such trials.^1–15^ Established and proposed accountability mechanisms include prospective registration and mandated results disclosure in public databases,^16^ authorship statements accepting responsibility for trial data and conclusions, lists of individual authors’ contributions to published articles, and disclosure of sources of funding and of investigators’ personal financial ties with for-profit entities.^17–22^ Another important mechanism that journals and others rely on to ensure the integrity and ethics of trials is the involvement of and oversight by an academic principal investigator (PI) who accepts responsibility for the design, conduct, and reporting of the trial.^23,24^

High-level statements about the PI emphasize the importance of the role in leading clinical trials. For example, the National Center for Advancing Translational Sciences (NCATS)^24^ defines the PI as “… the person(s) in charge of a clinical trial. … The [PI] prepares and carries out the clinical trial protocol (plan for the study). … The [PI] also analyzes the data and reports the results of the trial or grant research.” NCATS further explains that the PI

… is the researcher, usually a doctor or other medical professional, who leads the clinical research team and, along with the other members of the research team, regularly monitors study participants’ health to determine the study’s safety and effectiveness. A PI is primarily responsible for the preparation, conduct, and administration of a research grant, cooperative agreement, or other sponsored project in compliance with applicable laws and regulations and institutional policy governing the conduct of clinical research.

Although helpful, such statements lack granularity, hindering efforts to empirically validate the degree to which PIs in fact fulfill their responsibilities. Furthermore, although the PI’s job description has evolved as trials have become increasingly sponsor-initiated and multicenter,^25^ there has been no corresponding reevaluation of the nature of the PI’s role or of mechanisms of accountability for fulfilling it. For example, how should the PI be involved in, or assume responsibility for, activities such as selecting a study design, drafting the model consent form, reviewing eligibility or adverse event reports for individual participants, or framing the interpretation of study findings, and how do these norms vary depending on characteristics of the trial such as source of funding?

To address these questions, we conducted Delphi surveys of seven stakeholder-specific expert panels, including individuals representing PIs, journal editors, cooperative-group chairs, patient advocates, public funders, industry funders, and United States Food and Drug Administration (FDA) officials. We then surveyed PIs of multicenter clinical trials published in major general medical, oncology, cardiology, and psychiatry journals regarding the importance of PI responsibility for particular elements of clinical trials. We had two main objectives. First, we sought to clarify how stakeholders in the clinical trials enterprise view the roles and responsibilities of PIs, including how those views might differ by source of funding. Second, we aimed to establish a normative baseline against which the actual practices of PIs in leading particular trials—which we assessed in an independent survey to be reported separately—could be evaluated.

## Methods

### Overview

The study described here included two discrete phases. First, we conducted seven parallel Delphi panel surveys, each specific to a different stakeholder group, to explore how these stakeholders view the responsibilities of the PI of a multicenter trial. The panels were preceded by qualitative interviews with representatives of the same stakeholder groups to define relevant constructs for use in survey development. Next, we surveyed an international sample of PIs who had recently published a multicenter randomized controlled trial (RCT) to understand their normative views on PIs’ roles and responsibilities in such trials. We focused on multicenter RCTs because of their greater complexity and impact on practice and regulatory decisions as compared with single-arm and single-center trials.

### Participant selection

PIs were included as participants in both the Delphi panel and normative survey phases of the study. In contrast, members of the other six stakeholder groups participated in the Delphi panels alone.

We identified potentially eligible PIs based on their publication of a multicenter RCT testing a drug, device, or biological agent between October 2010 and July 2014 in one of 13 high-impact general medical, cardiology, oncology, or psychiatry journals (*New England Journal of Medicine*, *Lancet*, *JAMA*, *Annals of Internal Medicine*, *Journal of Clinical Oncology*, *Journal of the National Cancer Institute*, *Lancet Oncology*, *Circulation*, *Journal of the American College of Cardiology*, *American Journal of Psychiatry*, *Archives of General Psychiatry*, *Journal of Psychopharmacology*, and *Journal of Clinical Psychiatry*). Studies were excluded if the unit of randomization was not the individual patient, if any of the authors of the present report were also investigators on the study, or if the PI was on leave or deceased. For each article selected, we first contacted corresponding authors to confirm that they were the PIs of their respective studies; if a corresponding author identified another individual as the PI, we invited that individual instead. To invite PIs, we began with the articles in our sample frame with the earliest publication dates. No PI was invited to participate in more than one phase of the study.

We recruited participants for the journal-editor Delphi panel from among editors-in-chief of high-impact general medical, oncology, and cardiology journals (i.e. the same specialties from which we recruited PIs). Because no comprehensive sampling frames were available for the other five groups, we identified eligible individuals through a snowball approach that relied on personal contacts and on publicly available information.

### Delphi panels

The Delphi process is an established method used to collect and synthesize information from experts and to seek convergence in their views about a particular issue.^26,27^ It is most commonly operationalized as a sequence of questionnaires, beginning with a semi-structured instrument to identify topics or items to be addressed in later rounds. In subsequent rounds the questionnaires are increasingly structured, asking the panelists to re-rate each item along a series of dimensions relevant to the question at hand.

In this study, to define the relevant constructs and develop the surveys for the Delphi panels, we conducted semi-structured qualitative interviews with eight PIs of multicenter RCTs and two representatives from each of other six stakeholder groups. The interview guide included open-ended questions related to respondents’ perceptions of the PI’s main responsibilities, the domains included in the PI’s role, the nature of the PI’s responsibility for a trial, the concepts that best capture how PIs can exercise leadership, and interviewees’ views on whether PIs should have overall responsibility for the scientific validity, scientific integrity, and ethics of a trial. Interviews were conducted by an experienced qualitative researcher and averaged about 45 min. Coding was conducted using ATLAS.ti software. Participants were offered a $250 gift card in gratitude for their time.

After developing a survey based on the qualitative interviews, we conducted seven separate Delphi panels of up to 10 members, each representing one of the stakeholder groups (Supplementary Material Appendix 1: Table 1). Panelists responded to two rounds of survey questionnaires. Delphi surveys were conducted by mail in 2012–2013.

Panelists were first asked, “How important [is it] to the validity of a clinical trial, including both methodological quality and scientific integrity, that there be an identified academic PI with overall responsibility for the trial?” Responses used a 7-point ordinal scale (0 = not at all important; 6 = extremely important). This question was asked separately for publicly funded and for industry-funded trials. After responding to these two overall questions, panelists responded to 23 questions about PIs’ responsibilities for individual components of a publicly funded trial, followed by the same questions about an industry-funded trial. Each question asked about the extent to which the PI should be responsible for a specific task, decision, or product. For each item, respondents were also asked how a PI could acceptably exercise responsibility for that element. Response options included (1) leading a committee or group, (2) contributing to a committee or group, (3) delegating with substantial oversight, and (4) delegating with minimal oversight (Supplementary Material Appendix 2: Delphi Instrument).

In Round 2 of the Delphi panel, panelists were presented with their own previous ratings and with the aggregate responses of their stakeholder group panel. They were then provided the opportunity to change or reaffirm their previous response. Respondents were offered a $500 honorarium for completion of both rounds.

### Normative survey of PIs

Based on results from the Delphi panels, we developed a survey instrument to explore investigators’ normative views about the roles that PIs should play in leading multicenter randomized trials. The instrument mirrored that used in the Delphi panel exercise, with modifications to the response options regarding how PIs might acceptably exercise their leadership responsibility for elements of a trial. We eliminated one item (“adjudicating endpoint determinations for individual study participants”) based on feedback from the Delphi panelists that this task is often the responsibility of a blinded central review committee. The survey also included questions about the respondent, including advanced degrees, academic rank, work setting, gender, and total number of trials as PI (Supplementary Material Appendix 3: Normative Survey). Prior to survey launch, we conducted 13 cognitive interviews with a separate group of PIs to refine the instrument.

In 2014–2015, we solicited PIs of the earliest consecutive articles in our database who had not previously been invited to take part in a prior phase of the study to participate in the normative survey. Invitations were first sent via email with an embedded web-link to the survey. Two reminder emails were sent to non-respondents at weekly intervals. Nonrespondents to the three email invitations were sent a mailed invitation and paper version of the survey along with a prepaid return envelope. DatStat Illume (DatStat, Inc., Seattle, WA, USA), a web-based survey and data management package, supported the recruitment and data collection; study staff manually entered paper survey responses. PIs who completed the survey were offered a $50 gift card.

### Statistical analyses and sample size calculations

Analyses for both the Delphi panels and the normative survey were primarily descriptive. For the normative survey, we aimed for a sample size of 100 respondents to achieve 95% confidence intervals that were no wider than ±0.1 around the proportions who considered each task to be “important.” We also compared responses to paired questions about investigator responsibility for publicly versus industry-funded trials using Wilcoxon signed-rank tests.

### Ethics approval

This study was initially approved by the institutional review board (IRB) at the Dana-Farber Cancer Institute in Boston, MA. After the first author moved to the University of Pennsylvania, the study was approved by the IRB at that institution. Given the minimal-risk nature of the study, the IRBs waived the requirement for documentation of informed consent.

## Results

### Delphi panels

Of 149 individuals invited to participate in the seven Delphi panels, 52 (35%) initially agreed and 49/52 (94%) completed both rounds (Supplementary Material Appendix 1: Table 1). Panels ranged from 6 to 10 members. Most panelists were male (67%) and ages 55–69 (57%; Appendix 1: Table 2).

**Figure 1.**
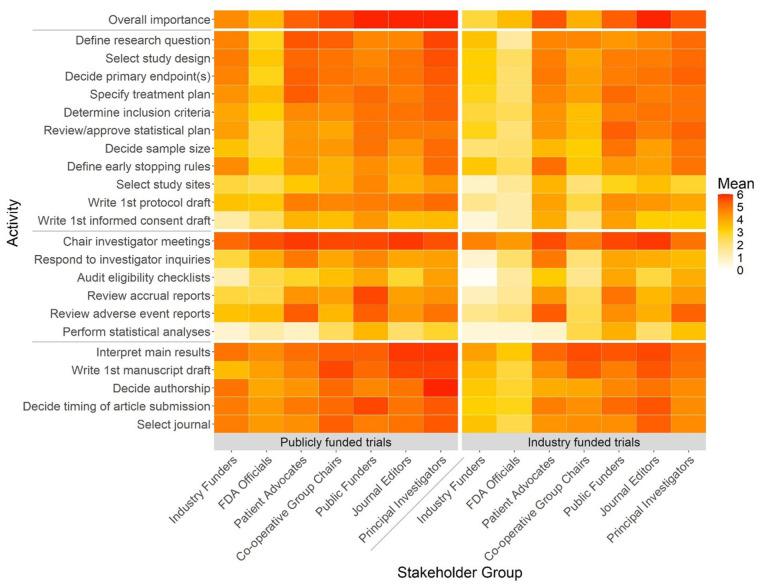
Delphi panel results: importance of principal investigator’s activities by stakeholder group, funder, and task. Overall importance was assessed by asking panelists, “Thinking about publicly/industry funded multicenter randomized controlled trials, how important is it to the validity of the trial, including both methodological quality and scientific integrity, that there be an identified academic PI with overall responsibility for the trial?” Responses were on a 7-point scale ranging from 0, “Not at all important,” to 6, “Extremely important.” The importance of PI responsibility for the specific tasks was assessed by asking, “Consider a publicly/industry funded RCT of a drug, device, or biological agent with over 200 participants from at least 10 sites in the PI’s home country. To what extent *should* the overall academic PI of the trial be responsible for [specific task]?” Responses were on a 7-point scale ranging from 0, “Not at all responsible,” to 6, “Completely responsible.”

When considering publicly funded multicenter RCTs, most stakeholder panels strongly endorsed the importance of involvement by an identified academic PI (Figure 1 and Supplementary Material Appendix 1: Table 3). They particularly endorsed the importance of academic PI involvement in tasks at the conception and design (e.g. defining the research question; specifying the treatment plan) and at the interpretation and dissemination (e.g. interpreting the main results; writing the first manuscript draft) stages. In contrast, they less strongly endorsed the importance of PI involvement in tasks at the trial conduct stage (e.g. auditing eligibility checklists, reviewing adverse event reports). Notably, panels representing industry funders and FDA officials viewed PI involvement in most tasks—especially those at the conception and design and the trial conduct stages—as less important than did panels representing other stakeholder groups.

Overall, panelists viewed involvement of an identified academic PI as less important when considering industry-funded as compared with publicly funded trials (Figure 1 and Supplementary Material Appendix 1: Table 3). The difference was especially striking for panels of industry funders and FDA officials, who viewed the involvement of an academic PI in industry-funded trials as modestly important both overall and with respect to virtually all listed tasks.

### Normative survey

**Figure 2.**
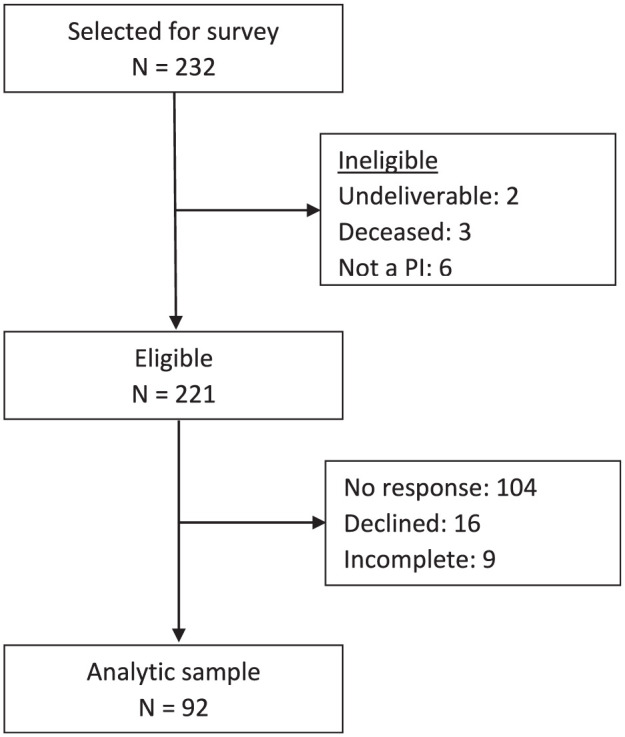
Consort diagram of normative survey participants.

Of 221 eligible PIs invited to participate in the normative survey, 92 (42%) responded (Figure 2). Response rates were lower among PIs of industry trials (33/102, 32%) than among PIs of non-industry or mixed-sponsor trials (39/72, 54% and 20/44, 45%, respectively; p = 0.013). Responders and non-responders did not differ significantly by specialty or by continental location (Supplementary Material Appendix 1: Table 4).

Most respondents were male (74%), had an MD degree or equivalent (92%), currently or previously held a faculty appointment (91%, of whom 78% had reached the rank of professor), and currently worked in an academic setting (84%). Respondents most commonly led trials in oncology (37%), cardiovascular medicine (18%), and infectious disease (15%; Table 1).

**Table 1.** Characteristics of normative survey respondents (N = 92).

	N (%)
Sex	
Male	68 (74)
Female	21 (23)
Unknown	3 (3)
Advanced degree(s)	
MD, with or without another degree	85 (92)
Other	5 (6)
Unknown	2 (2)
Ever had a faculty appointment^a^	
Yes	83 (91)
No	7 (8)
Highest current or past academic faculty rank^b^	
Professor or equivalent	65 (78)
Other	16 (19)
Current work setting	
University or academic medical center	77 (84)
Pharmaceutical, biotechnology, or medical device company	4 (4)
Other	8 (9)
Unknown	3 (3)
Study specialty	
Oncology	34 (37)
Cardiovascular	17 (18)
Infectious disease	14 (15)
Endocrinology and metabolism	5 (5)
Pulmonary	5 (5)
Psychiatry	4 (4)
Other^c^	13 (14)
Location	
North America	45 (49)
Europe	41 (45)
Other^d^	6 (7)
Total number of trials for which respondent was the overall PI/study chair^a^	
≤2	20 (22)
3–5	23 (25)
6–10	16 (17)
≥11	30 (33)
	N (%)
Total number of *randomized* trials for which respondent was the overall PI/study chair^a^	
≤2	26 (28)
3–5	26 (28)
6–10	16 (17)
≥11	21 (23)
Total number of *multicenter* trials for which respondent was the overall PI/study chair^a^	
≤2	32 (35)
3–5	22 (24)
6–10	16 (17)
≥11	19 (21)
Total number of *industry-funded* trials for which respondent was the overall PI/study chair^a^	
≤2	57 (62)
3–5	8 (9)
6–10	13 (14)
≥11	10 (11)
Total number of *publicly funded* trials for which respondent was the overall PI/study chair^a^	
≤2	39 (42)
3–5	27 (29)
6–10	10 (11)
≥11	13 (14)

a Numbers do not add to 92 due to missing responses.

b Responses were missing from 2/83 participants with current or past academic appointments.

c Includes critical care (3), neonatology (2), urology (2), gynecology (1), hematology (1), neurology (1), pathology (1), rheumatology (1), and transplantation (1).

d Other includes Africa (1), Asia (2), Australia (2), and South America (1).

Approximately half had served as PI for ≥6 trials. When asked about specific types of trials, 40% had served as PI for ≥6 randomized trials, 38% for ≥6 multicenter trials, 25% for ≥6 industry-funded trials, and 25% for ≥6 publicly funded trials.

**Figure 3.**
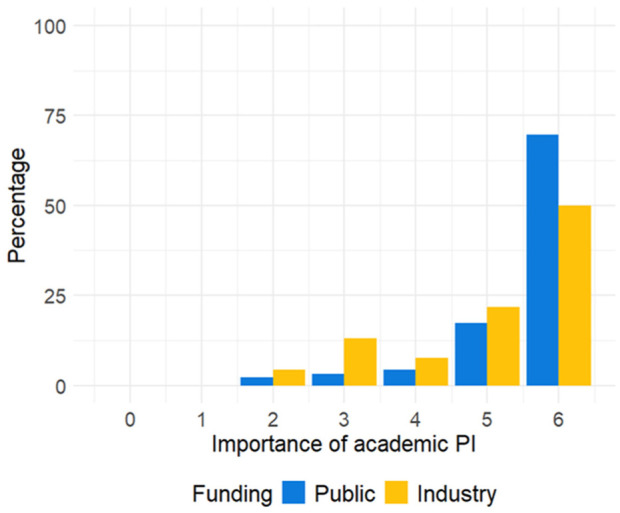
Principal investigators’ views regarding the importance of an academic PI to the validity of a multicenter randomized controlled trial, by study funder (N = 89). Respondents were asked, “How important it is to the validity of a trial, including both methodological quality and scientific integrity, that there be an identified academic PI/Study Chair with overall responsibility for a publicly funded/industry-funded multicenter RCT?” Response options were on a 7-point scale from 0, “Not at all important,” to 6, “Extremely important.” P-value, comparing responses for public versus industry funder, was <0.001. Responses were missing from three respondents.

Respondents generally viewed the involvement of an academic PI as important to the validity of both publicly and industry-funded multicenter RCTs; median responses to both questions were 6 (“extremely important”). Despite these generally high ratings, however, many respondents viewed academic PIs as playing a considerably more important role in publicly funded than in industry-funded trials (p < 0.001; Figure 3).

**Figure 4.**
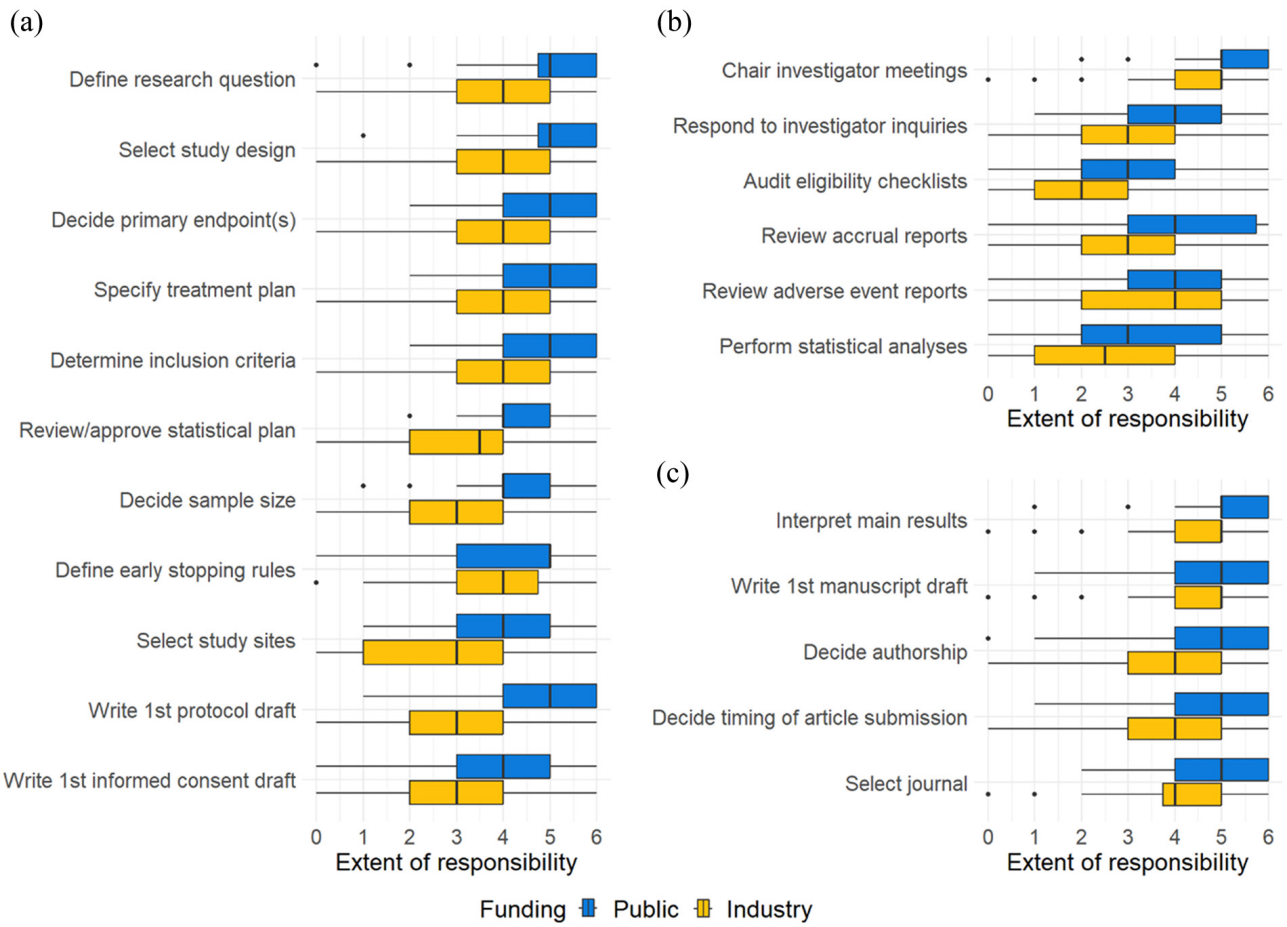
Principal investigators’ views regarding PI responsibility for specific tasks in multicenter randomized controlled trials, by study funder and task (N = 92). (a) Design, (b) conduct, and (c) dissemination. Question stem for all items: “To what extent should the overall PI/Study Chair of this trial exercise responsibility for …?” Response options were on a 7-point scale from 0, “No responsibility,” to 6, “Complete responsibility.” P-values from Wilcoxon signed-rank test ≤0.001 for all paired comparisons of funding type.

Respondents were next asked about the importance of academic PI responsibility for 22 specific tasks. When considering publicly funded trials, most respondents believed that academic PIs should have complete or near-complete responsibility for most tasks, especially those involving conception and design (e.g. defining the research question, selecting the study design) and those involving interpretation and dissemination (e.g. interpreting the main results, deciding authorship). Responsibility scores were lower for tasks at the trial conduct stage (e.g. responding to inquiries from investigators, auditing eligibility checklists). When asked about the importance of an academic PI’s involvement in industry-funded trials, median responsibility scores were generally at least one point lower than for the corresponding tasks in publicly funded trials (p ≤ 0.001 for all paired comparisons; Figure 4 and Supplementary Material Appendix 1: Figure 1).

**Figure 5.**
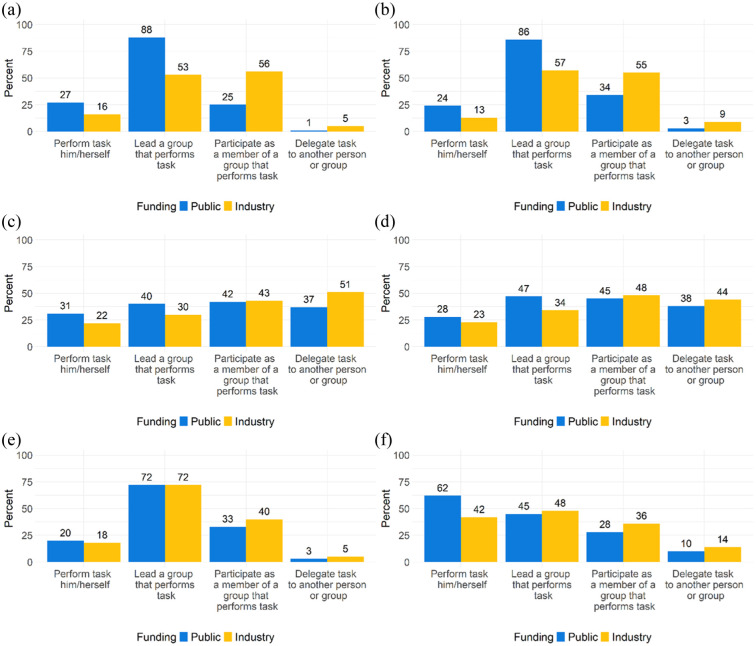
Principal investigators’ views regarding how PIs should exercise their responsibility, by study funder and representative task. (a) Define research question, (b) specify treatment plan, (c) respond to investigator inquiries, (d) review adverse event reports, (e) interpret main results, and (f) write first manuscript draft. Responses are shown for six representative tasks, across the study’s life cycle from conception to reporting. Supplementary Material Appendix Figure 1 includes responses for the full set of 22 tasks. Respondents could check more than one option, so percentages do not sum to 100.

Finally, we asked how PIs might acceptably exercise their responsibility for carrying out the 22 tasks. Respondents generally favored exercising responsibility by leading or participating as a member of a group, with fewer endorsing sole PI responsibility. For only four tasks—writing the first consent form draft, chairing investigator meetings, responding to inquiries from site investigators (both industry trials only), auditing eligibility checklists, and performing statistical analyses—did most respondents endorse delegating responsibility. In general, respondents endorsed more PI-centered mechanisms of exercising responsibility when considering publicly funded as compared with industry-funded trials. Figures 5(a)–(f) show responses to representative items; Supplementary Material Appendix 1, Figure 1 includes the complete set of tasks.

## Discussion

Academic PIs are widely viewed as linchpins of integrity and accountability for clinical trials, especially in the setting of industry-funded trials whose design, conduct, and reporting might be influenced by commercial imperatives.^23,24,28^ Little is known, however, about whether participants in the clinical research enterprise endorse the norm that the “buck stops with the PI” or about whether or how they distinguish among the specific roles and responsibilities of the PI in such trials. To address these gaps, we elucidated the perspectives of PIs and other relevant groups regarding the role of the PI in multicenter RCTs.

Our data show that, common pronouncements notwithstanding, stakeholders do not uniformly view academic PIs as critical to the leadership of clinical trials. In particular, based on responses to our Delphi survey, stakeholders who bring an industry or FDA perspective are less likely than other groups to endorse the importance of academic PIs in leading trials, both overall and with respect to specific trial-related tasks. Equally important, and contrary to statements that emphasize the academic PI as a bulwark of integrity in industry-funded trials, stakeholders view leadership by academic PIs as less central to industry-funded trials than to trials funded by public sponsors. These findings challenge widely held assumptions about the nature of the PI’s role and raise pressing questions about leadership and accountability in the clinical trials enterprise.

Our data also show that stakeholders—both Delphi panelists and survey respondents—see PIs as bearing greater responsibility for some trial-related tasks than for others. In particular, respondents generally consider PIs to be most responsible for tasks related to the design of the trial as well as those related to its analysis, interpretation, and dissemination, whereas they consider PIs to be less responsible for tasks related to the day-to-day conduct of ongoing trials. Furthermore, across all tasks included in our survey, respondents view PI responsibility as less important in the context of industry-funded trials than in that of publicly funded trials. These differences between industry-funded and publicly funded trials are apparent in tasks as fundamental as defining the research question, selecting the study design, drafting the protocol, interpreting the main results, and writing the first draft of the study manuscript. Respondents similarly are more likely to find participation as a member of a group or delegating the task to be an acceptable method of exercising responsibility when considering industry-funded as compared with publicly funded trials; this difference aligns with our overarching finding that stakeholders view leadership by an academic PI as less central to the former than to the latter type of trial.

In light of authoritative statements holding that academic PIs are central to trial integrity and accountability, the fact that respondents de-emphasize PIs’ importance in leading industry-funded as compared with publicly funded trials may seem surprising. This distinction may reflect structural requirements. For example, the National Institutes of Health hold the PI—often the grant or contract recipient—responsible for design, analysis, and reporting of the trial^24^; in contrast, the FDA assigns overall responsibility for the design, conduct, and analysis of trials that it regulates to the sponsor (usually a medical products company), while holding investigators responsible for trial conduct at each site rather than for the integrity of the study as a whole.^29,30^ The observation by Sekeres et al.^31^ that information about a trial’s scientific leadership was missing from almost 60% of registry listings of trials sponsored by industry, but was almost always present in registry listings of trials sponsored by non-industry funders, is consistent with our panelists’ and respondents’ views that an academic PI is more central to the former than to the latter group of trials.

That participants in the clinical research enterprise do not uniformly endorse the central role of academic PIs has important ethical and practical implications for the conduct and integrity of trials. First, it suggests the need for a better understanding of how PIs actually exercise leadership, as the assumption that PIs can be held accountable for trials may not be accurate. This need for transparency is particularly acute in the context of industry-funded trials, where the focus on the PI may obscure the roles of company employees who actually lead the trials. Indeed, when company employees direct trials, journal editors should insist that they publicly accept responsibility by serving as corresponding authors on trial reports rather than expecting an academic investigator to fulfill that function. Finally, if PIs are often less involved in the leadership of trials than is commonly believed, other mechanisms of accountability, such as sharing of protocols, data, and analyses, require greater emphasis. Rigorous scrutiny by regulators is an especially important accountability mechanism; notably, most industry-funded trials are subject to such scrutiny, whereas trials conducted in academia and those funded by other sources do not usually receive such oversight.

Several limitations of our study deserve mention. First, we sampled PIs based on their leadership of studies published in high-impact general medical or specialty journals; their perspectives may therefore not generalize to the universe of PIs, including those whose trials are published in lower-tier journals and those in specialties other than oncology, cardiology, and psychiatry. Second, because of the small numbers of respondents in each Delphi panel, and because there is no systematic way to identify stakeholders other than PIs for inclusion, panelists’ views may not be representative of all members of their respective stakeholder groups. In addition, although Delphi methods have the virtue of avoiding dominance by influential individuals, they may overestimate consensus and mask minority views.^32^ Third, our findings may be affected by response bias, as 33% of those invited to join the Delphi panels and 40% of those invited to join the normative survey ultimately took part. Importantly, response rates to the normative survey were lowest among PIs of industry-funded trials, suggesting that the views of this group may be underrepresented in our data. Fourth, our observations may not generalize to single-center or non-randomized trials or to trials that test interventions other than drugs, devices, or biological agents. Fifth, we do not address PI responsibility for a number of important tasks, such as their role in making trial data available or responding to queries after study publication. Sixth, although we aimed to assess respondents’ normative views about how PIs *should* lead clinical trials, some may have had their perceptions of the actual practices of PIs in mind as they answered our survey.

An additional limitation is that data for this study were collected from 2012 to 2015. However, given the limited attention to the issues we studied and the lack of relevant regulatory changes, it is unlikely that there have been interval changes in the norms regarding the role of the PI that might have altered our major findings. The few relevant events since that time include a federal mandate to submit study results to ClinicalTrials.gov, an obligation that attaches either to the sponsor or to the PI so long as the PI

is responsible for conducting the trial, has access to and control over the data from the clinical trial, has the right to publish the results of the trial, and has the ability to meet all of the [relevant] requirements … for the submission of clinical trial information.^33^

In addition, building on recommendations from the Presidential Commission for the Study of Bioethical Issues, in 2013, the Secretary’s Advisory Committee on Human Research Protections proposed adding a section on investigator responsibilities to the regulations governing federally funded human subjects research.^34,35^ These recommendations, which focused on PIs’ qualifications, their role in human subjects protection, and their documentation responsibilities, were not adopted into regulation. These developments are insufficient to change long-standing norms regarding leadership of clinical trials during the intervening years.

In conclusion, we find striking discrepancies between widely held assumptions and authoritative statements about the importance of academic PIs in leading clinical trials—especially those sponsored by industry—and the views of diverse stakeholders in the clinical trials enterprise. These discrepancies suggest that PIs may not always exercise robust responsibility for the clinical trials that they ostensibly lead and, therefore, that they may not universally fulfill the accountability function that is generally ascribed to them. If so, alternative means of assuring clinicians and patients of the quality and integrity of clinical trials are urgently needed.

## Supplemental Material

sj-docx-1-ctj-10.1177_17407745261417337 – Supplemental material for Stakeholder views about the responsibilities of principal investigators in multicenter randomized controlled trials Supplemental material, sj-docx-1-ctj-10.1177_17407745261417337 for Stakeholder views about the responsibilities of principal investigators in multicenter randomized controlled trials by Steven Joffe, Elizabeth F Bair, Katharine A Gleason, Deborah E Sellers, Sarah A McGraw, Cary P Gross, Donna T Chen, Eric G Campbell and Michelle M Mello in Clinical Trials

sj-docx-2-ctj-10.1177_17407745261417337 – Supplemental material for Stakeholder views about the responsibilities of principal investigators in multicenter randomized controlled trials Supplemental material, sj-docx-2-ctj-10.1177_17407745261417337 for Stakeholder views about the responsibilities of principal investigators in multicenter randomized controlled trials by Steven Joffe, Elizabeth F Bair, Katharine A Gleason, Deborah E Sellers, Sarah A McGraw, Cary P Gross, Donna T Chen, Eric G Campbell and Michelle M Mello in Clinical Trials

sj-pdf-3-ctj-10.1177_17407745261417337 – Supplemental material for Stakeholder views about the responsibilities of principal investigators in multicenter randomized controlled trials Supplemental material, sj-pdf-3-ctj-10.1177_17407745261417337 for Stakeholder views about the responsibilities of principal investigators in multicenter randomized controlled trials by Steven Joffe, Elizabeth F Bair, Katharine A Gleason, Deborah E Sellers, Sarah A McGraw, Cary P Gross, Donna T Chen, Eric G Campbell and Michelle M Mello in Clinical Trials
