# Supplementary material for: Stakeholder views about the responsibilities of principal investigators in multicenter randomized controlled trials
Source: Clin Trials. 2026 Feb 23;23(3):325–35. doi: 10.1177/17407745261417337 (PMC12944536; doi:10.1177/17407745261417337)

**APPENDIX 1**

Appendix Table 1. Participation in Delphi panel, by stakeholder group

Appendix Table 2. Demographic characteristics of round 2 Delphi panel participants with analyzable data (N = 49)

Appendix Table 3. Delphi panel ratings of the importance of principal investigator’s activities by stakeholder group, funder, and task – mean values

Appendix Table 4. Characteristics of responders and nonresponders to principal investigator survey

Appendix Figure 1. Principal investigators’ views regarding how PIs should exercise their responsibility, by study funding source

Appendix Table 1. Participation in Delphi panel, by stakeholder group

| Stakeholder group | Invited to participate | Completed  round 1 | Completed  round 2 |
| --- | --- | --- | --- |
| Principal investigators | 22 | 7 | 7 |
| Journal editors | 20 | 8 | 6 |
| Public funders | 17 | 6 | 6 |
| Co-operative group chairs | 19 | 7 | 6 |
| Patient advocates | 15 | 10 | 10 |
| Industry funders | 27 | 7 | 7 |
| FDA officials | 29 | 7 | 7 |
| Total | 149 | 52 | 49 |

Appendix Table 2. Demographic characteristics of round 2 Delphi panel participants with analyzable data (N = 49)

|  | N (%) |
| --- | --- |
| Sex |  |
| Male | 33 (67) |
| Female | 16 (33) |
| Age |  |
| 40-54 | 19 (39) |
| 55-69 | 28 (57) |
| Unknown | 2 (4) |
| Race |  |
| White | 42 (86) |
| Other^*^ | 5 (10.0) |
| Unknown | 2 (4) |
| Ethnicity |  |
| Hispanic/Latino | 2 (4) |
| Non-Hispanic | 45 (92) |
| Unknown | 2 (4) |

^*^ Other includes 2 Black or African American, 1 Asian, 1 biracial/multiracial, and 1 other.

Appendix Table 3. Delphi panel ratings of the importance of principal investigator’s activities by stakeholder group, funder, and task – mean values

| Publicly funded trials | | | | | | | |
| --- | --- | --- | --- | --- | --- | --- | --- |
| Activity | Principal Investigators | Journal Editors | Public Funders | Co-operative Group Chairs | Patient Advocates | Industry Funders | FDA Officials |
| Overall importance | 6 | 6 | 6 | 5.7 | 5.3 | 4.6 | 3.6 |
| Define research question | 5.7 | 4.7 | 4.7 | 5.3 | 5.5 | 4.7 | 3 |
| Select study design | 5.6 | 5 | 4.7 | 5 | 5.2 | 4.9 | 3.3 |
| Decide primary endpoint(s) | 5.4 | 5 | 4.8 | 5 | 5.3 | 4.7 | 3 |
| Specify treatment plan | 5.3 | 4.8 | 5.2 | 4.8 | 5.4 | 4.4 | 3.6 |
| Determine inclusion criteria | 5.3 | 5 | 5 | 4.5 | 4.6 | 4 | 3.1 |
| Review/approve statistical plan | 4.9 | 4.8 | 5 | 4 | 4.3 | 4.1 | 2.7 |
| Decide sample size | 5.1 | 4.3 | 5 | 4.3 | 4.4 | 3.4 | 2.7 |
| Define early stopping rules | 5.1 | 4 | 4.5 | 3.8 | 4.4 | 4.6 | 3.1 |
| Select study sites | 4.3 | 3.8 | 4.7 | 3.8 | 3.3 | 2.7 | 2.4 |
| Write 1st protocol draft | 5.1 | 4.8 | 4.8 | 4.7 | 4.8 | 3.4 | 3.3 |
| Write 1st informed consent draft | 3.6 | 3.5 | 4.3 | 3.5 | 3.7 | 1.3 | 2.3 |
| Respond to investigator inquiries | 4.1 | 4 | 4.7 | 4 | 4.9 | 2.7 | 3.9 |
| Audit eligibility checklists | 4.1 | 2.8 | 4 | 3.5 | 2.9 | 1.1 | 2.6 |
| Review accrual reports | 4.4 | 4.2 | 5.7 | 4.2 | 4.4 | 2.7 | 2.6 |
| Review adverse event reports | 5 | 4.3 | 5.3 | 3.7 | 5.4 | 3.4 | 3.6 |
| Perform statistical analyses | 2.9 | 2.2 | 3.7 | 2.3 | 0.9 | 0.7 | 1.3 |
| Interpret main results | 5.9 | 5.8 | 5.3 | 5.3 | 5.1 | 5 | 4.6 |
| Write 1st manuscript draft | 5.7 | 5.7 | 5.2 | 5.7 | 4.8 | 3.6 | 4.1 |
| Decide authorship | 6 | 5 | 4.7 | 5.2 | 4.4 | 5 | 4 |
| Decide timing of article submission | 5.4 | 5 | 5.7 | 5.2 | 4.9 | 4.9 | 4.3 |
| Select journal | 5.4 | 5 | 4.8 | 5.3 | 4.5 | 4.8 | 4.3 |
| Chair investigator meetings | 5.6 | 5.8 | 5.7 | 5.7 | 5.8 | 5.2 | 5.6 |

Appendix Table 3 (continued). Delphi panel ratings of the importance of principal investigator’s activities by stakeholder group, funder, and task – mean values

| Industry funded trials | | | | | | | |
| --- | --- | --- | --- | --- | --- | --- | --- |
| Activity | Principal Investigators | Journal Editors | Public Funders | Co-operative Group Chairs | Patient Advocates | Industry Funders | FDA Officials |
| Overall importance | 5.4 | 6 | 5.3 | 3.8 | 5.5 | 2.7 | 3.6 |
| Define research question | 5.1 | 4.7 | 4.3 | 4.7 | 4.7 | 3.4 | 1.4 |
| Select study design | 5.1 | 4.8 | 4.8 | 4 | 4.8 | 3.1 | 2.3 |
| Decide primary endpoint(s) | 5.3 | 5 | 4.8 | 4.2 | 4.9 | 3.1 | 2.1 |
| Specify treatment plan | 5 | 4.8 | 5.2 | 4.2 | 4.7 | 3 | 2.1 |
| Determine inclusion criteria | 5 | 5 | 4.8 | 3.5 | 4.4 | 2.7 | 2.4 |
| Review/approve statistical plan | 5.3 | 4.8 | 5.3 | 3.5 | 4.4 | 3 | 2 |
| Decide sample size | 5 | 4.2 | 5 | 3.2 | 3.6 | 2 | 2.1 |
| Define early stopping rules | 5 | 4.2 | 4.3 | 3.3 | 5.1 | 3.3 | 2.4 |
| Select study sites | 2.9 | 3.5 | 3 | 2 | 3.7 | 0.9 | 1.6 |
| Write 1st protocol draft | 4 | 4.3 | 4.5 | 2.7 | 4.1 | 1.7 | 1.3 |
| Write 1st informed consent draft | 3.1 | 3.2 | 4.2 | 1.8 | 3.9 | 0.6 | 1.3 |
| Chair investigator meetings | 5 | 5.8 | 5.7 | 4.8 | 5.6 | 4.7 | 4.3 |
| Respond to investigator inquiries | 3.6 | 3.8 | 4 | 2 | 4.9 | 0.7 | 2.1 |
| Audit eligibility checklists | 3.9 | 2.7 | 4 | 1.7 | 3.2 | 0.1 | 1.4 |
| Review accrual reports | 4.3 | 3.7 | 5 | 2.3 | 4.3 | 1 | 1.6 |
| Review adverse event reports | 5.3 | 3.8 | 4.5 | 2.5 | 5.4 | 1.7 | 2 |
| Perform statistical analyses | 3.4 | 2.2 | 3.8 | 2.6 | 0.8 | 0.6 | 0.6 |
| Interpret main results | 5.1 | 5.7 | 5.5 | 5.6 | 5.2 | 4.1 | 3.3 |
| Write 1st manuscript draft | 5 | 5.5 | 4.8 | 5.4 | 4.5 | 3.6 | 2.7 |
| Decide authorship | 4.6 | 5 | 4.7 | 4 | 3.9 | 3.3 | 2.9 |
| Decide timing of article submission | 4.6 | 5.5 | 5.2 | 4.5 | 4.8 | 3.1 | 3 |
| Select journal | 4.6 | 5.3 | 4.5 | 4.5 | 4.4 | 3.4 | 2.6 |

Appendix Table 4. Characteristics of responders and non-responders to principal investigator survey

|  | Respondent  (N = 92) | Non-respondent  (N = 129) | p-value^*^ |
| --- | --- | --- | --- |
| Specialty |  |  | 0.074 |
| Cardiology | 17 (18) | 35 (27) |  |
| Oncology | 34 (37) | 52 (40) |  |
| Psychiatry | 4 (4) | 10 (8) |  |
| Other | 37 (40) | 32 (25) |  |
|  |  |  |  |
| Funding^†^ |  |  | 0.013 |
| Industry | 33 (36) | 69 (53) |  |
| Non-Industry | 39 (42) | 33 (26) |  |
| Mixed | 20 (22) | 22 (17) |  |
|  |  |  |  |
| Location of corresponding author |  |  | 0.406 |
| Africa | 1 (1) | 0 (0) |  |
| Asia | 2 (2) | 6 (5) |  |
| Australia | 2 (2) | 0 (0) |  |
| Europe | 41 (45) | 56 (43) |  |
| North America | 45 (49) | 66 (51) |  |
| South America | 1 (1) | 1 (1) |  |

^*^Significance tests for specialty and funding were chi-square tests. The significance test for location was a Fisher’s exact test.

^†^No funding source was specified in articles associated with 5 non-responders.

Note: Numbers in parentheses are column percents.

Appendix Figure 1: Principal investigators’ views regarding how PIs should exercise their responsibility, by study funding source


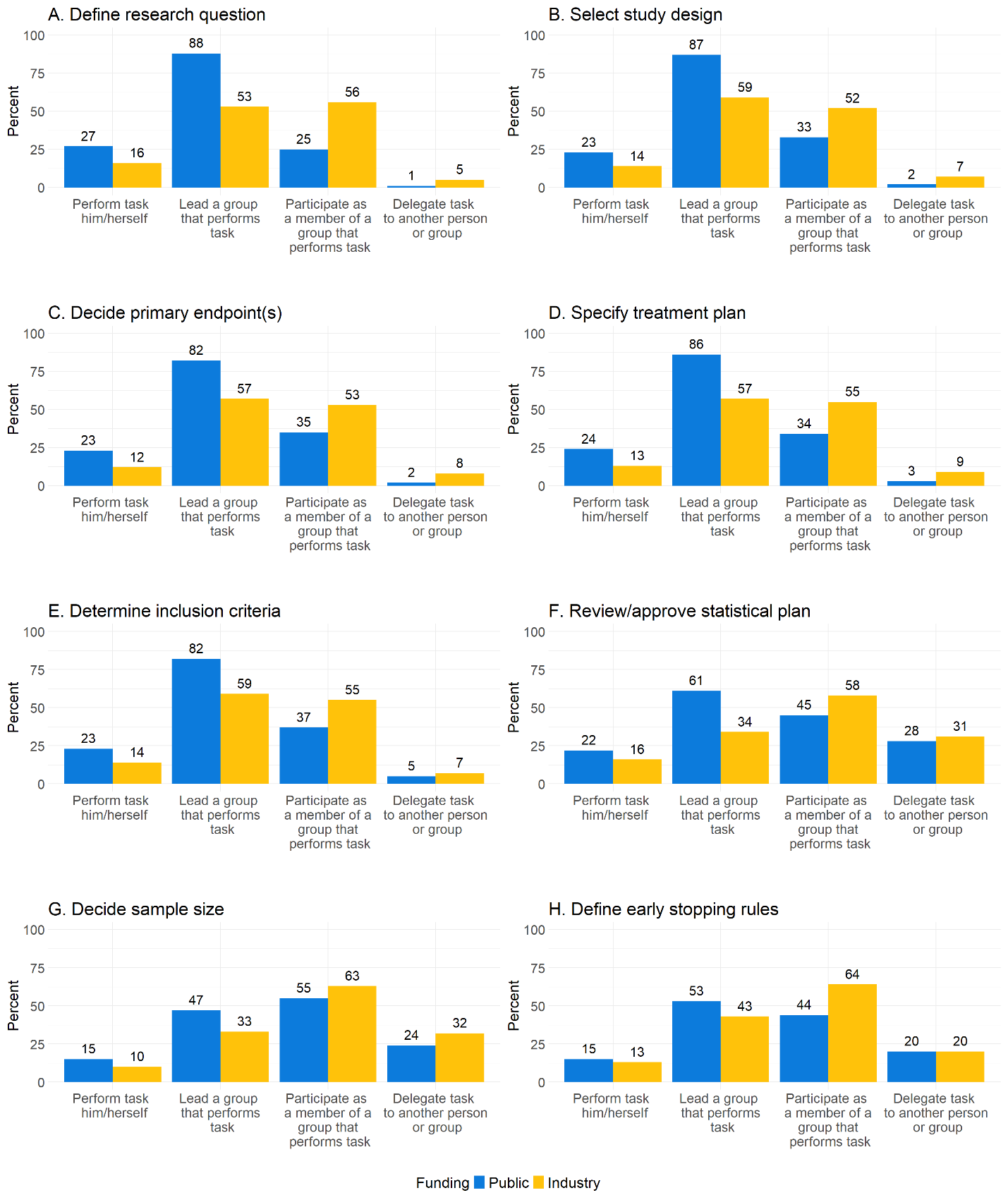


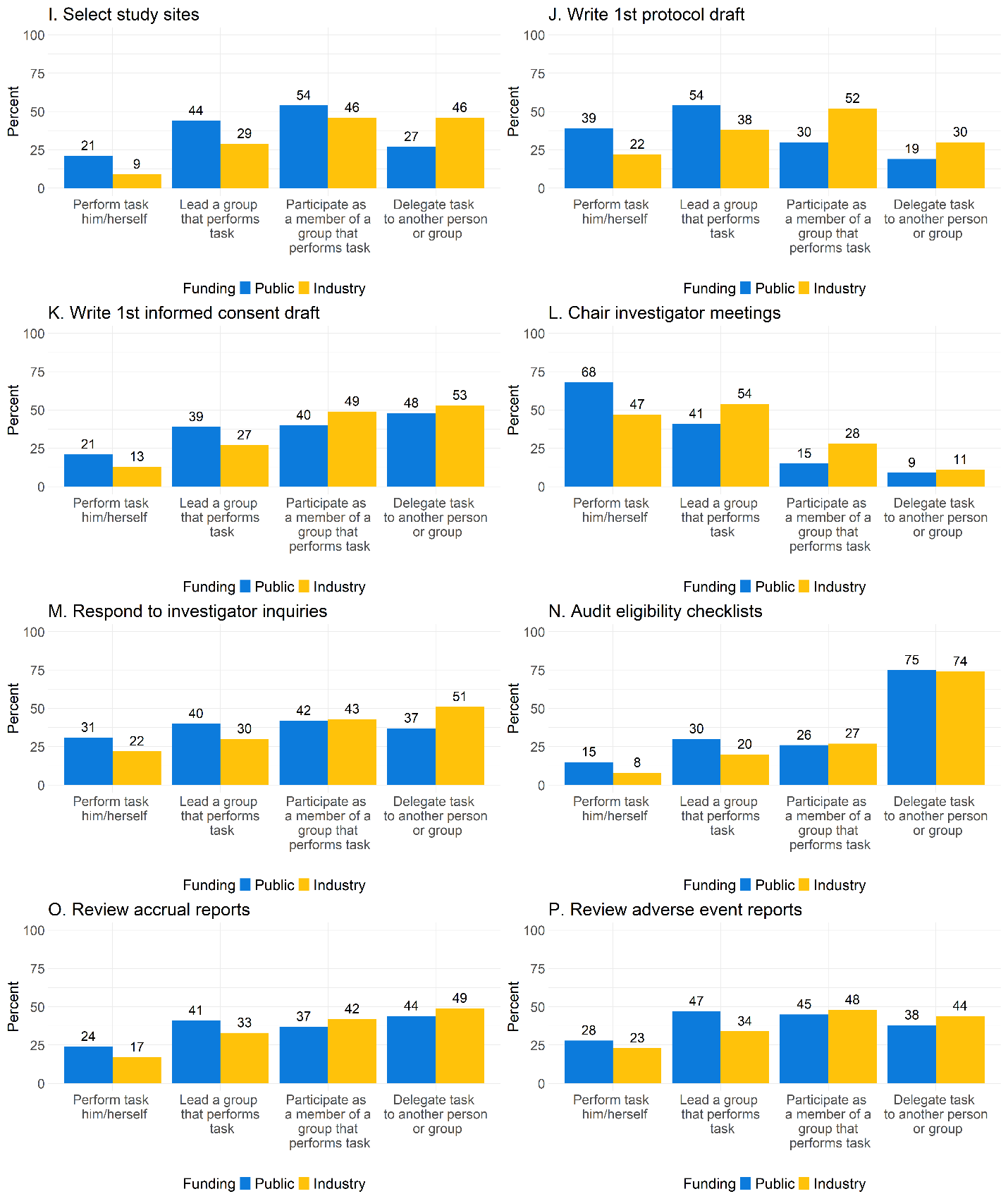


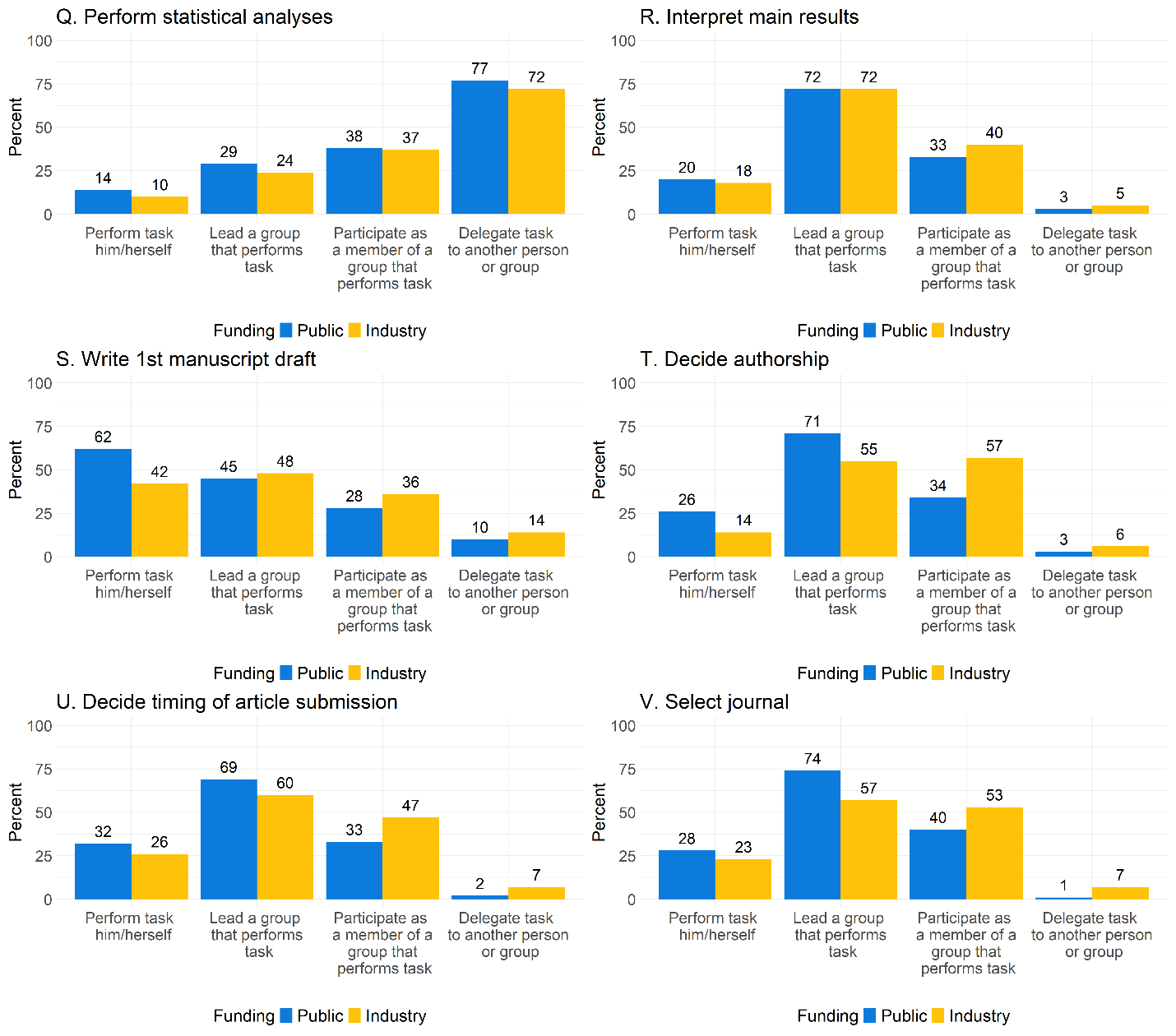

Supplement: sj-docx-1-ctj-10.1177_17407745261417337 – Supplemental material for Stakeholder views about the responsibilities of principal investigators in multicenter randomized controlled trials [file sj-docx-1-ctj-10.1177_17407745261417337.docx]
