# Supplementary material for: Stakeholder views about the responsibilities of principal investigators in multicenter randomized controlled trials
Source: Clin Trials. 2026 Feb 23;23(3):325–35. doi: 10.1177/17407745261417337 (PMC12944536; doi:10.1177/17407745261417337)
Supplement: sj-docx-2-ctj-10.1177_17407745261417337 – Supplemental material for Stakeholder views about the responsibilities of principal investigators in multicenter randomized controlled trials [file sj-docx-2-ctj-10.1177_17407745261417337.docx]

**Appendix 2:**

Delphi Instrument

**1. Thinking about *publicly-funded* multicenter RCTs, how important is it to the validity of the trial, including both methodological quality and scientific integrity, that there be an identified academic PI with overall responsibility for the trial?**

| **Not at all**  **Important** |  |  |  |  |  | **Extremely**  **Important** |
| --- | --- | --- | --- | --- | --- | --- |
| 0 | 1 | 2 | 3 | 4 | 5 | 6 |
|  |  |  |  |  |  |  |

**2. Thinking about *industry-funded* multicenter RCTs, how important is it to the validity of the trial, including both methodological quality and scientific integrity, that there be an identified academic PI with overall responsibility for the trial?**

| **Not at all**  **Important** |  |  |  |  |  | **Extremely**  **Important** |
| --- | --- | --- | --- | --- | --- | --- |
| 0 | 1 | 2 | 3 | 4 | 5 | 6 |
|  |  |  |  |  |  |  |

| **3. Consider a *publicly-funded* RCT of a drug, device, or biologic agent with over 200 participants from at least 10 sites in the PI’s home country:** | | | | | | | | | | | |
| --- | --- | --- | --- | --- | --- | --- | --- | --- | --- | --- | --- |
|  | **A. To what extent *should* the overall academic PI of this trial be responsible for …?** | | | | | | | **B. What are acceptable ways for the overall academic PI of this trial to exercise responsibility for…?**  **(Check all that Apply)** | | | |
|  | **Not at all**  **Responsible** | |  |  |  | **Completely**  **Responsible** | |  |  |  |  |
|  | **0** | **1** | **2** | **3** | **4** | **5** | **6** | **Lead**  **Committee** | **Contribute**  **Committee** | **Delegate**  **Substantial**  **Oversight** | **Delegate**  **Minimal**  **Oversight** |
| 1. defining the research questions |  |  |  |  |  |  |  |  |  |  |  |
| 1. selecting the study design |  |  |  |  |  |  |  |  |  |  |  |
| 1. deciding on the study’s primary endpoint(s) |  |  |  |  |  |  |  |  |  |  |  |
| 1. specifying the treatment plan for each study arm |  |  |  |  |  |  |  |  |  |  |  |
| 1. determining the inclusion and exclusion criteria for study participants |  |  |  |  |  |  |  |  |  |  |  |
| 1. reviewing and approving the statistical analysis plan |  |  |  |  |  |  |  |  |  |  |  |
| 1. making the final decision about sample size |  |  |  |  |  |  |  |  |  |  |  |
| 1. defining the early stopping rules |  |  |  |  |  |  |  |  |  |  |  |
| 1. selecting the study sites |  |  |  |  |  |  |  |  |  |  |  |
| 1. writing the first draft of the study protocol |  |  |  |  |  |  |  |  |  |  |  |
| 1. writing the first draft of the model informed consent form |  |  |  |  |  |  |  |  |  |  |  |
| 1. responding to inquiries from investigators at local sites |  |  |  |  |  |  |  |  |  |  |  |
| 1. auditing completed eligibility checklists for individual study participants |  |  |  |  |  |  |  |  |  |  |  |
| 1. reviewing accrual monitoring reports |  |  |  |  |  |  |  |  |  |  |  |
| 1. reviewing adverse event reports for individual study participants |  |  |  |  |  |  |  |  |  |  |  |
| 1. adjudicating endpoint determinations for individual study participants |  |  |  |  |  |  |  |  |  |  |  |
| 1. performing statistical analyses |  |  |  |  |  |  |  |  |  |  |  |
| 1. deciding how the study’s main results should be interpreted |  |  |  |  |  |  |  |  |  |  |  |
| 1. writing the first draft of the manuscript reporting the primary study results |  |  |  |  |  |  |  |  |  |  |  |
| 1. deciding who will be an author on the manuscript reporting the primary study results |  |  |  |  |  |  |  |  |  |  |  |
| 1. deciding when the manuscript reporting the primary study results will be submitted for publication |  |  |  |  |  |  |  |  |  |  |  |
| 1. selecting the journal to which the manuscript reporting the primary study results will be submitted |  |  |  |  |  |  |  |  |  |  |  |
| 1. chairing investigator meetings and conference calls |  |  |  |  |  |  |  |  |  |  |  |

| **4. Consider an *industry-funded* RCT of a drug, device, or biologic agent with over 200 participants from at least 10 sites in the PI’s home country, and with an *academic PI who was invited by the trial sponsor to serve in that role*** | | | | | | | | | | | |
| --- | --- | --- | --- | --- | --- | --- | --- | --- | --- | --- | --- |
|  | **A. To what extent *should* the overall academic PI of this trial be responsible for …?** | | | | | | | **B. What are acceptable ways for the overall academic PI of this trial to exercise responsibility for…?**  **(Check all that Apply)** | | | |
|  | **Not at all**  **Responsible** | |  |  |  | **Completely**  **Responsible** | |  |  |  |  |
|  | **0** | **1** | **2** | **3** | **4** | **5** | **6** | **Lead**  **Committee** | **Contribute**  **Committee** | **Delegate**  **Substantial**  **Oversight** | **Delegate**  **Minimal**  **Oversight** |
| 1. defining the research questions |  |  |  |  |  |  |  |  |  |  |  |
| 1. selecting the study design |  |  |  |  |  |  |  |  |  |  |  |
| 1. deciding on the study’s primary endpoint(s) |  |  |  |  |  |  |  |  |  |  |  |
| 1. specifying the treatment plan for each study arm |  |  |  |  |  |  |  |  |  |  |  |
| 1. determining the inclusion and exclusion criteria for study participants |  |  |  |  |  |  |  |  |  |  |  |
| 1. reviewing and approving the statistical analysis plan |  |  |  |  |  |  |  |  |  |  |  |
| 1. making the final decision about sample size |  |  |  |  |  |  |  |  |  |  |  |
| 1. defining the early stopping rules |  |  |  |  |  |  |  |  |  |  |  |
| 1. selecting the study sites |  |  |  |  |  |  |  |  |  |  |  |
| 1. writing the first draft of the study protocol |  |  |  |  |  |  |  |  |  |  |  |
| 1. writing the first draft of the model informed consent form |  |  |  |  |  |  |  |  |  |  |  |
| 1. responding to inquiries from investigators at local sites |  |  |  |  |  |  |  |  |  |  |  |
| 1. auditing completed eligibility checklists for individual study participants |  |  |  |  |  |  |  |  |  |  |  |
| 1. reviewing accrual monitoring reports |  |  |  |  |  |  |  |  |  |  |  |
| 1. reviewing adverse event reports for individual study participants |  |  |  |  |  |  |  |  |  |  |  |
| 1. adjudicating endpoint determinations for individual study participants |  |  |  |  |  |  |  |  |  |  |  |
| 1. performing statistical analyses |  |  |  |  |  |  |  |  |  |  |  |
| 1. deciding how the study’s main results should be interpreted |  |  |  |  |  |  |  |  |  |  |  |
| 1. writing the first draft of the manuscript reporting the primary study results |  |  |  |  |  |  |  |  |  |  |  |
| 1. deciding who will be an author on the manuscript reporting the primary study results |  |  |  |  |  |  |  |  |  |  |  |
| 1. deciding when the manuscript reporting the primary study results will be submitted for publication |  |  |  |  |  |  |  |  |  |  |  |
| 1. selecting the journal to which the manuscript reporting the primary study results will be submitted |  |  |  |  |  |  |  |  |  |  |  |
| 1. chairing investigator meetings and conference calls |  |  |  |  |  |  |  |  |  |  |  |
