# Supplementary material for: Stakeholder views about the responsibilities of principal investigators in multicenter randomized controlled trials
Source: Clin Trials. 2026 Feb 23;23(3):325–35. doi: 10.1177/17407745261417337 (PMC12944536; doi:10.1177/17407745261417337)
Supplement: sj-pdf-3-ctj-10.1177_17407745261417337 – Supplemental material for Stakeholder views about the responsibilities of principal investigators in multicenter randomized controlled trials [file sj-pdf-3-ctj-10.1177_17407745261417337.pdf]

Appendix 3

Normative Survey: The Role of the Principal Investigator in Multicenter Trials

The Role of the Principal Investigator in Multicenter Trials

Principal Investigator / Study Chair Survey

a project of

Perelman School of Medicine
UNIVERSITY of PENNSYLVANIA

funded by
The National Cancer Institute
The National Institutes of Health

October, 2014
©University of Pennsylvania 2014

Thank you for agreeing to participate in our brief survey. The survey will ask your opinion, as an experienced Principal Investigator (PI)/Study Chair, about how much responsibility the overall PI/Study Chair of a multi-center randomized controlled trial (RCT) should have for various elements of the trial. We will also ask you what, in your opinion, are the acceptable ways for the overall PI/Study Chair to exercise that responsibility. We plan to survey 100 PIs/Study Chairs of multi-center RCTs for this study.

Your participation is voluntary. You may refuse to participate at any time. You may also skip any questions that you do not want to answer. We don't foresee any significant risks. The main risk is loss of confidentiality. However, our procedures should minimize the risk of loss of confidentiality. More information about this study can be found in the Study Description that is included in this packet.

After you complete the survey, we will send you a $50 gift card/check to thank you for your participation. If you are unable to accept this gift card/check, please let us know by checking the appropriate box at the end of the survey. We hope you will nevertheless agree to take part in this important study.

The survey should take about 15-20 minutes to complete.

STATEMENT OF CONSENT

Have you been PI/Study Chair on 1 or more multicenter randomized trials of a drug, medical device, or biological agent?

❑ Yes, → do you consent to participate in the survey?

- ❑ Yes
- ❑ No, please return the blank survey in the envelope provided, or fax this page to 1-215-573-3036, or scan it to joffes@upenn.edu

❑ No, please return the blank survey in the envelope provided, or fax this page to 1-215-573-3036, or scan it to joffes@upenn.edu

[insert UIN]

The questions in this survey ask about your views regarding the roles that an overall academic Principal Investigator (PI)/Study Chair should play in publicly-funded and in industry-funded multicenter randomized controlled trials (RCTs). We are interested in the overall academic PI, who is sometimes called the Study Chair or Chief Investigator, not in the PIs at individual study sites.

In industry-funded trials, the overall academic PI/Study Chair is external to the company sponsoring the trial. This person is typically a physician in academic medicine who was invited by the trial funder to serve as the overall academic PI/Study Chair. Not all industry-funded trials have an overall academic PI/Study Chair.

As you answer these questions, for both publicly-funded and industry-funded trials, please think about a Phase III trial of a drug, device, or biological agent that involves over 200 subjects from at least 10 sites.

1. How important is it to the validity of a trial, including both methodological quality and scientific integrity, that there be an identified academic PI/Study Chair with overall responsibility for…

a. a **publicly-funded** multicenter RCT

Not at all Important ▼ | Please circle one number. | Extremely Important ▼

0 **1** **2** **3** **4** **5** **6**

b. an **industry-funded** multicenter RCT

Not at all Important ▼ | Please circle one number. | Extremely Important ▼

0 **1** **2** **3** **4** **5** **6**

Every trial, whether publicly- or industry-funded, involves a series of specific decisions, tasks, and products. For each of these decisions, tasks, and products we are interested in two questions: A) to what extent the overall academic PI/Study Chair of the trial should, in your opinion, exercise responsibility for that specific study task, decision, or product; and B) your views on the acceptable ways for the overall academic PI/Study Chair to exercise his or her responsibility, if any, for that task, decision, or product.

For the tasks for which you believe that the PI has some responsibility (that is, a response of 1 to 6 in Part A), the second question of each pair (Part B) asks for your views on the acceptable ways for the overall academic PI/Study Chair to exercise his/her responsibility for that task, decision, or product. There can be more than one acceptable way, so please check all that apply.

If you answer 0 for part A of an item, please skip part B and continue with the next item.

2. Consider a *publicly-funded* RCT of a drug, device, or biologic agent with over 200 subjects from at least 10 sites:

A. To what extent should the overall academic PI/Study Chair of this trial exercise responsibility for…?

For each question, circle a number for Part A.

If you answer 0 for part A of an item, please skip part B and continue with the next item.

B. Which are acceptable ways for the overall academic PI/Study Chair of this trial to exercise responsibility for…?

Check all that apply

	No Responsibility ▼						Complete Responsibility ▼	Performing this task him/herself ▼	Leading a group that performs this task ▼	Participating as a member of a group that performs this task ▼	Delegating this task to another person or group ▼
a. defining the research question	0	1	2	3	4	5	6	❑	❑	❑	❑
b. selecting the study design	0	1	2	3	4	5	6	❑	❑	❑	❑
c. deciding on the study's primary endpoint(s)	0	1	2	3	4	5	6	❑	❑	❑	❑
d. specifying the treatment plan for each study arm	0	1	2	3	4	5	6	❑	❑	❑	❑
e. determining the inclusion and exclusion criteria for study subjects	0	1	2	3	4	5	6	❑	❑	❑	❑
f. reviewing and approving the statistical analysis plan	0	1	2	3	4	5	6	❑	❑	❑	❑

Continued from previous page

2. Consider a *publicly-funded* RCT of a drug, device, or biologic agent with over 200 subjects from at least 10 sites:

A. To what extent should the overall academic PI/Study Chair of this trial exercise responsibility for…?

For each question, circle a number for Part A.

If you answer 0 for part A of an item, please skip part B and continue with the next item.

B. Which are acceptable ways for the overall academic PI/Study Chair of this trial to exercise responsibility for…?

Check all that apply

	No Responsibility ▼						Complete Responsibility ▼	Performing this task him/herself ▼	Leading a group that performs this task ▼	Participating as a member of a group that performs this task ▼	Delegating this task to another person or group ▼
g. making the final decision about sample size	0	1	2	3	4	5	6	❑	❑	❑	❑
h. defining the early stopping rules	0	1	2	3	4	5	6	❑	❑	❑	❑
i. selecting the study sites	0	1	2	3	4	5	6	❑	❑	❑	❑
j. writing the first draft of the study protocol	0	1	2	3	4	5	6	❑	❑	❑	❑
k. writing the first draft of the model informed consent form	0	1	2	3	4	5	6	❑	❑	❑	❑
l. responding to inquiries from investigators at local sites	0	1	2	3	4	5	6	❑	❑	❑	❑
m. auditing completed eligibility checklists for individual study subjects	0	1	2	3	4	5	6	❑	❑	❑	❑
n. reviewing accrual monitoring reports	0	1	2	3	4	5	6	❑	❑	❑	❑
o. reviewing adverse event reports for individual study subjects	0	1	2	3	4	5	6	❑	❑	❑	❑

Continued from previous page

2. Consider a *publicly-funded* RCT of a drug, device, or biologic agent with over 200 subjects from at least 10 sites:

A. To what extent should the overall academic PI/Study Chair of this trial exercise responsibility for…?

For each question, circle a number for Part A.

If you answer 0 for part A of an item, please skip part B and continue with the next item.

B. Which are acceptable ways for the overall academic PI/Study Chair of this trial to exercise responsibility for…?

Check all that apply

	No Responsibility ▼						Complete Responsibility ▼	Performing this task him/herself ▼	Leading a group that performs this task ▼	Participating as a member of a group that performs this task ▼	Delegating this task to another person or group ▼
p. performing statistical analyses	0	1	2	3	4	5	6	❑	❑	❑	❑
q. deciding how the study's main results should be interpreted	0	1	2	3	4	5	6	❑	❑	❑	❑
r. writing the first draft of the manuscript reporting the primary study results	0	1	2	3	4	5	6	❑	❑	❑	❑
s. deciding who will be an author on the manuscript reporting the primary study results	0	1	2	3	4	5	6	❑	❑	❑	❑
t. deciding when the manuscript reporting the primary study results will be submitted for publication	0	1	2	3	4	5	6	❑	❑	❑	❑
u. selecting the journal to which the manuscript reporting the primary study results will be submitted	0	1	2	3	4	5	6	❑	❑	❑	❑
v. chairing investigator meetings and conference calls	0	1	2	3	4	5	6	❑	❑	❑	❑

For this next set of questions please think about an industry-funded trial in which the overall academic PI/Study Chair is a physician in academic medicine who was invited by the trial funder to serve in that role. This PI, who is external to the company funding the trial, is sometimes referred to as the Study Chair and/or Chief Investigator. (Please note that we are not asking about the clinical investigator who is responsible for the study at one particular site).

3. Consider an *industry-funded* RCT of a drug, device, or biologic agent with over 200 subjects from at least 10 sites:

A. To what extent should the overall academic PI/Study Chair of of this trial exercise responsibility for…?

B. Which are acceptable ways for the overall academic PI/Study Chair of this trial to exercise responsibility

For each question, circle a number for Part A.

If you answer 0 for part A of an item, please skip part B and continue with the next item.

Check all that apply

	**No Responsibility** ▼						**Complete Responsibility** ▼	**Performing this task him/herself** ▼	**Leading a group that performs this task** ▼	**Participating as a member of a group that performs this task** ▼	**Delegating this task to another person or group** ▼
a. defining the research question	**0**	**1**	**2**	**3**	**4**	**5**	**6**	❑	❑	❑	❑
b. selecting the study design	**0**	**1**	**2**	**3**	**4**	**5**	**6**	❑	❑	❑	❑
c. deciding on the study's primary endpoint(s)	**0**	**1**	**2**	**3**	**4**	**5**	**6**	❑	❑	❑	❑
d. specifying the treatment plan for each study arm	**0**	**1**	**2**	**3**	**4**	**5**	**6**	❑	❑	❑	❑
e. determining the inclusion and exclusion criteria for study subjects	**0**	**1**	**2**	**3**	**4**	**5**	**6**	❑	❑	❑	❑
f. reviewing and approving the statistical analysis plan	**0**	**1**	**2**	**3**	**4**	**5**	**6**	❑	❑	❑	❑
g. making the final decision about sample size	**0**	**1**	**2**	**3**	**4**	**5**	**6**	❑	❑	❑	❑

Continued from previous page

3. Consider an *industry-funded* RCT of a drug, device, or biologic agent with over 200 subjects from at least 10 sites:

A. To what extent should the overall academic PI/Study Chair of this trial exercise responsibility for…?

For each question, circle a number for Part A.

If you answer 0 for part A of an item, please skip part B and continue with the next item.

B. Which are acceptable ways for the overall academic PI/Study Chair of this trial to exercise responsibility for…?

Check all that apply

	No Responsibility ▼						Complete Responsibility ▼	Performing this task him/herself ▼	Leading a group that performs this task ▼	Participating as a member of a group that performs this task ▼	Delegating this task to another person or group ▼
h. defining the early stopping rules	0	1	2	3	4	5	6	❑	❑	❑	❑
i. selecting the study sites	0	1	2	3	4	5	6	❑	❑	❑	❑
j. writing the first draft of the study protocol	0	1	2	3	4	5	6	❑	❑	❑	❑
k. writing the first draft of the model informed consent form	0	1	2	3	4	5	6	❑	❑	❑	❑
l. responding to inquiries from investigators at local sites	0	1	2	3	4	5	6	❑	❑	❑	❑
m. auditing completed eligibility checklists for individual study subjects	0	1	2	3	4	5	6	❑	❑	❑	❑
n. reviewing accrual monitoring reports	0	1	2	3	4	5	6	❑	❑	❑	❑
o. reviewing adverse event reports for individual study subjects	0	1	2	3	4	5	6	❑	❑	❑	❑

Continued from previous page

3. Consider an *industry-funded* RCT of a drug, device, or biologic agent with over 200 subjects from at least 10 sites:

A. To what extent should the overall academic PI/Study Chair of this trial exercise responsibility for…?

B. Which are acceptable ways for the overall academic PI/Study Chair of this trial to exercise responsibility for…?

For each question, circle a number for Part A.

If you answer 0 for part A of an item, please skip part B and continue with the next item.

Check all that apply

	No Responsibility ▼						Complete Responsibility ▼	Performing this task him/herself ▼	Leading a group that performs this task ▼	Participating as a member of a group that performs this task ▼	Delegating this task to another person or group ▼
p. performing statistical analyses	0	1	2	3	4	5	6	❑	❑	❑	❑
q. deciding how the study's main results should be interpreted	0	1	2	3	4	5	6	❑	❑	❑	❑
r. writing the first draft of the manuscript reporting the primary study results	0	1	2	3	4	5	6	❑	❑	❑	❑
s. deciding who will be an author on the manuscript reporting the primary study results	0	1	2	3	4	5	6	❑	❑	❑	❑
t. deciding when the manuscript reporting the primary study results will be submitted for publication	0	1	2	3	4	5	6	❑	❑	❑	❑
u. selecting the journal to which the manuscript reporting the primary study results will be submitted	0	1	2	3	4	5	6	❑	❑	❑	❑
v. chairing investigator meetings and conference calls	0	1	2	3	4	5	6	❑	❑	❑	❑

The following questions are about you.

Education and Training

4. Which of the following advanced degrees do you hold?
(Please check *all that apply* and indicate the year you received each degree)

Degree	Year Received
☐ Medical Doctor (i.e., MD or equivalent)	________
☐ PhD (or equivalent) → What field? ____________________________	________
☐ Other (please specify: ______________________) → What field? _________________	________

5. Have you ever had an academic faculty appointment?
(*check one*)

- ☐ No → *skip to Question 6*
- ☐ Yes, current
- ☐ Yes, past

5a. Highest current or past academic rank (check one)

- ☐ Professor or equivalent
- ☐ Associate Professor or equivalent
- ☐ Assistant Professor or equivalent
- ☐ Instructor/Lecturer or equivalent
- ☐ Other (please specify: ______________________________)

6. What is your current work setting?

☐ University or academic medical center

☐ Pharmaceutical, biotechnology or medical device company

☐ Government agency

☐ Other (please specify: ____)

7. What is your gender?

☐ Male

☐ Female

Your Experience as an Overall PI/Study Chair

Please check one box for each item to indicate the approximate number of clinical trials (i.e., intervention studies testing drugs, devices or biological agents) for which you have served as the overall PI/Study Chair.

	<3	3-5	6-10	11-20	>20
8. Total number of clinical trials as the overall PI/Study Chair (not a site PI/Study Chair)?	☐	☐	☐	☐	☐
9. Among clinical trials listed in item 8 above, approximately how many were ***randomized*** trials?	☐	☐	☐	☐	☐
10. Among clinical trials listed in item 8 above, approximately how many were ***multi-center*** trials?	☐	☐	☐	☐	☐
11. Among clinical trials listed in item 8 above, approximately how many were entirely or mainly ***funded by industry***?	☐	☐	☐	☐	☐
12. Among clinical trials listed in item 8 above, approximately how many were entirely or mainly ***funded by government sources***?	☐	☐	☐	☐	☐

Your Comments

13. Please share with us any additional thoughts or comments you have related to the roles and responsibilities of the PI/Study Chair of multi-center randomized controlled trials.

We welcome both comments on specific questions from the survey as well as your more general thoughts on this topic.

Honorarium

14. Are you able to accept the $50 honorarium for completing this survey?

- ☐ Yes → Please return the enclosed form "Preferred Mailing Address for Gift Card or Check" in the mailing envelope with your survey, fax it to 1-215-573-3036, or scan it to joffes@upenn.edu.
- ☐ No

Thank You for Completing the Survey

Please return the completed survey and the "Preferred Mailing Address for Gift Card or Check" form to:

Steven Joffe, MD, MPH
Perelman School of Medicine
University of Pennsylvania
Department of Medical Ethics and Health Policy
3401 Market Street, Suite 320
Philadelphia, PA 19104-3319
joffes@upenn.edu
fax: 215-573-3036
